# Deep Learning-Based Classification of Spoken English Digits

**DOI:** 10.1155/2022/3364141

**Published:** 2022-09-28

**Authors:** Jane Oruh, Serestina Viriri

**Affiliations:** School of Mathematics, Statistics and Computer Science, University of KwaZulu-Natal, Durban, South Africa

## Abstract

Classification of isolated digits is the basic challenge for many speech classification systems. While a lot of work has been carried out on spoken languages, only limited research work on spoken English digit data has been reported in the literature. The paper proposes an intelligent-based system based on deep feedforward neural network (DFNN) with hyperparameter optimization techniques, an ensemble method; random forest (RF), and a regression method; gradient boosting (GB) for the classification of spoken digit data. The paper investigates different machine learning (ML) algorithms to determine the best method for the classification of spoken English digit data. The DFNN classifier outperformed the RF and GB classifiers on the public benchmark spoken English digit data and achieved 99.65% validation accuracy. The outcome of the proposed model performs better compared to existing models with only traditional classifiers.

## 1. Introduction

Speech is a means of communicating information from one or more speakers to one or more listeners. The speech produced by a speaker carries data in the form of signals, which are being transported from the mouth of the speaker to the ear of the listener. Speech is made up of sequences of phonemes, which are uttered at an average rate of approximately 12 phonemes per second [1]. Speech communication has become a predominant model for information exchange and social interaction among humans. Speech recognition is an emerging technology in the area of natural language processing (NLP) by Jurafsky and Martin [2].

Classification of speech is one of the most essential issues in speech processing [3]. Classification is the procedure of labeling a given set of data into classes. The process is conducted on organized data as well as on unorganized data. It starts with predicting the class of given data points which are known as targets, labels, or categories. The essence of classification predictive modeling is to map input values, *x*, to category *y*, output values [4] using a mathematical function. Classification of isolated digits is the basic challenge for many speech classification systems. Limited studies have been conducted on the classification of the English digit data.

The challenge with spoken digit recognition is a result of the following: (1) the spoken digits are of short acoustic duration, normally a few seconds of speech; (2) Some digits are acoustically identical to each other [5], Kopparapu and Rao [6]. The importance of this challenge has led several authors to research how to enhance digit recognition for different languages, which includes English [7], Portuguese [8], Arabic [9], and Mandarin [10].

The model proposes an intelligent-based system that will make use of a deep feedforward neural network (DFNN) with hyperparameter optimization techniques, an ensemble method; random forest (RF), and a regression method; gradient boosting (GB) for the classification of the spoken English digit data. The proposed DFNN performance was evaluated using hyperparameter optimization techniques such as adaptive moment estimation (Adam) optimization algorithm and stochastic gradient descent (SGD) optimization algorithm. Adam's optimization algorithm showed a better result than the classical SGD optimization algorithm. Optimization is a method of finding the best value of some function or model. Optimization for test cases aims at minimizing the number of test cases while delivering the best fault coverage [11]. Short-term Fourier transform (STFT) was used to extract features from the audio data before performing one-hot encoding to produce the class label.

In our previous work, the proposed model used only DFNN with optimization techniques for the classification of the spoken English digit data [12], but in this work, the classification approach has been extended to use RF, GB, and DFNN for the classification of the spoken English digit data. The essence is to compare the performances of three different machine learning (ML) algorithms and to determine the best approach amongst them for classification purposes. The result from our experiment shows that DFNN is the best classification method compared to RF and GB.

Contributions to this research are as follows:

1. A brief review of RF, GB, and DFNN methods and techniques is presented.
2. The paper investigates three (3) different ML algorithms to determine the best approach for the classification of spoken English digit data.

The rest of the paper is arranged in this form: Section 2 considers recent speech classification techniques and their achievements.  Section 3 gives a comprehensive description of the proposed ML algorithms, methods, and techniques for the proposed model.  Section 4 discusses the experiments, model training, and experimental results and presents a relative analysis of the proposed model. Finally, Section 5 presents the conclusion.

## 2. Related Works

Speech classification in recent times has left several authors with the challenge of having to investigate the best method for achieving optimum accuracy.

The model proposes a novel approach that can be used to classify Bengali spoken digits using the convolutional neural network (CNN) [13]. The voice recordings of ten (10) individuals were classified considering gender, dialects, and age groups. The result of the classification accuracy is 98.37%, which shows the credibility of the proposed approach. The result here is bounded for Bengali spoken digits.

The work proposes a speech pathology recognition system that will automatically analyze the voice system of patients [14]. NN and deep learning methods were used for the classification of speech signals, to distinguish between a voice signal that is normal or pathological. The Levenberg Marquardt algorithm was used for classifying voice signals, whereas the restricted Boltzmann machine algorithm was used to implement the deep learning classification of the voice signals. The restricted Boltzmann machine algorithm shows an accuracy of 98.00% compared to the Levenberg Marquardt algorithm with 92.00% accuracy. The accuracy of the proposed model can be improved when tested with the other ML algorithms during network training.

The proposed model combines lexicon-based and machine learning methods for the prediction of hate speech, based on sentiment analysis [15]. The emotional facts found in the text assisted in improving the accuracy of hate speech detection, from 41.00% in the previous work to 80.64% on the test result. The proposed model could use deep learning optimization techniques alongside the lexicon-based and ML methods to improve hate speech accuracy.

A progressive rendering of a real-time speech emotion recognition application using the AlexNet image classification network was proposed in [16]. The baseline approach shows the result of 82.00% accuracy on the Berlin emotional speech (EMO-DB) data. The proposed model could not attain a high accuracy even with the AlexNet pretrained network.

The model proposed in [17] introduces a new multimodal deep learning framework that instinctively extracts features from textual-acoustic data for speech intention classification. The proposed system was tested in a real medical setting to serve as a reference for future research. The model achieved an average accuracy of 83.10% when 6 different intentions were detected. The model proposed here has performed better than existing models that used manufactured features. The proposed model accuracy is not very high.

The study in [18] studied a good deal of speech classification algorithms. A comparative analysis of five classification algorithms was conducted. Based on the result of investigations, a multilayer perceptron with 93.00% accuracy by the Robust scaler method was proposed. Achieving such accuracy for the proposed method is restricted to using the Robust scaler method to scale the multilayer perceptron. A deep feedforward multilayer perceptron was proposed in this work, and the accuracy was 99.65%.

In [19], a deep CNN was used to advance Pashto isolated digit recognition. Mel frequency cepstral coefficients (MFCC) were used in extracting features from the speech signal. The result shows an accuracy of 84.17% for testing, which is equivalent to a 7.32% improvement in comparison with existing works. The proposed approach is edged in Pashto isolated digit recognition.

In a recent study on Dari one-word speech recognition, CNN was used in recognition of the isolated words in Dari speech [20] using deep learning algorithms. MFCC was used for feature extraction during training. The test result shows 88.2% accuracy, which reveals that the proposed method predicts visualized words with high accuracy during training. The use of other deep learning techniques for the analysis of the Dari speech can improve the model accuracy.

Marcolla et al. [21] proposed a new approach known as “lie detection” for speech classification using a voice stress analysis method. The authors employed the long short-term memory (LSTM) network to analyze and classify a person's speech as authentic or not. The best neural network model in the proposed method showed a precision accuracy of 72.5%. The result is scientifically remarkable for such problems as voice stress analysis, which implies that it is possible to find patterns in the voice of people who are under stress. The precision accuracy is considerably not high and could be improved.

A multiclass classification was conducted on the spoken English digit dataset using support vector machine (SVM), K-nearest neighbor (KNN), and random forest (RF) [22]. RF performed better than SVM and KNN. With 10% testing data, 97.50% accuracy was obtained. Using ML methods with hyperparameter optimization techniques as proposed in this work yielded a high accuracy of 99.65% on the same dataset.

A speech classification module was developed in [23] that will identify the appropriate speech for generating a medical report. The evaluation of the proposed model was performed using CNNs and LSTMs. Several parameters were tested and the performance of the model on different speaker features was examined. CNNs show 92.41% validation accuracy on 2709 speech segment data and are more thriving than LSTM networks. The proposed model could possibly be evaluated using different types of machine learning algorithms to obtain optimal validation accuracy, as shown in this work.

Sánchez–Hevia et al. [24] analyzed the performances of various deep neural networks (DNNs) for age estimation and differentiating gender from speech in interactive voice response (IVR) systems. The results of their experiment indicate good results for all the types of networks for gender classification, but combining CNNs and temporal convolutional networks (CTCN) gives a better result for the age group classification. Their best systems showed about 80% and 70% for precision and recall, respectively. Precision and recall accuracy is relatively high.

The proposed research in [25] used an RF and SVM classifier on 200 images of the standard Odia database for simulation. The simulation result shows 96.3%, 98.2%, 88.9%, and 93.6% accuracy on the Odia character and the Odia numerical database, respectively. The result is bounded in the Odia database.

The study by [26] presented an automated recognition system that will accurately classify authentic and forged signatures for offline signature verification. The proposed model was compared with six pretrained CNNs architecture based on transfer learning (TL) across a collection of publicly available signature samples. The outcome of their experiment shows 88% accuracy on the proposed model compared to other related networks and can be approved as a prototype for offline signature verification. The proposed model's accuracy could be improved using different machine learning algorithms to obtain optimal accuracy.

Sethy et al. [27] proposed an automated hybrid system for handwritten character recognition. The proposed model was tested on three benchmark datasets; Odia characters, Bangla numerals, and Odia numerals. The overall performance of handwritten Odia characters is 99.01% and 98.1%, Odia numerals are 98.6% and 97.6%, and Bangla numerals are 97.6% and 96.3%, respectively. The result analysis shows the best performance for least-square (LS)-SVM compared to RF. The performance of the proposed system is high but can be improved.

## 3. Methods and Techniques

**Figure 1:**
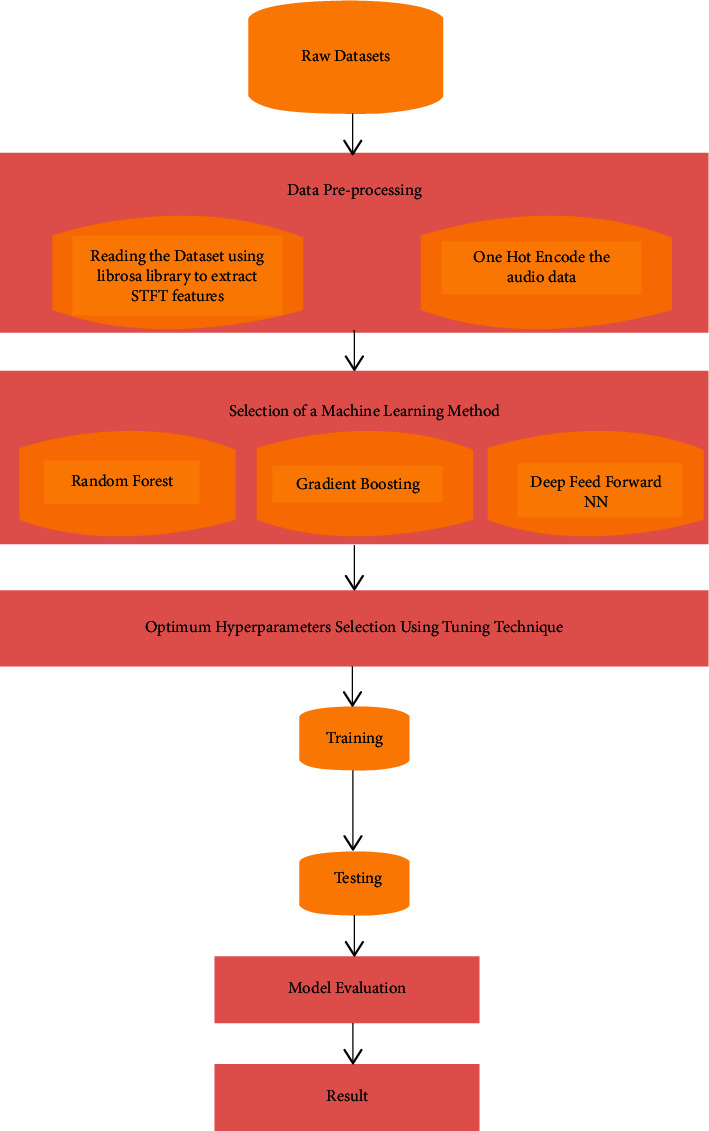
Flowchart of the proposed ML-based model.

The section initiates a progressive step in developing the proposed model. The proposed method for classifying the English digit data using ML algorithms; DFNN, RF, and GB includes the steps depicted in Figure 1. Data preprocessing is a step in which the raw data is transformed, or encoded, to bring it to a state that is appropriate for a machine or a deep learning model, and it is the first and pivotal step while creating a model.

### 3.1. Reading Dataset

The audio data is read using the library “Librosa.” STFT features were extracted from the audio data [28]. The main idea behind the STFT feature extraction is to break up the longer time signals into smaller fragments of the same length and then compute the Fourier transform independently on each of the smaller fragments. The continuous form of STFT is expressed as

(1) $$\begin{array}{c} STFTx\left(t\right)\left(\tau ,\omega \right)\equiv X\left(\tau ,\omega \right)=\int_{-\infty }^{\infty }x\left(t\right)\omega \left(t-\tau \right)e^{-j\omega t}dt\text{.} \end{array}$$

The discrete form of STFT is conveyed as

(2) $$\begin{array}{c} X\left(n,\omega \right)=\overset{\infty }{\underset{m=-\infty }{\sum }}x\left(m\right)\omega \left(n-m\right)e^{-j\omega m}\text{.} \end{array}$$

**Figure 2:**
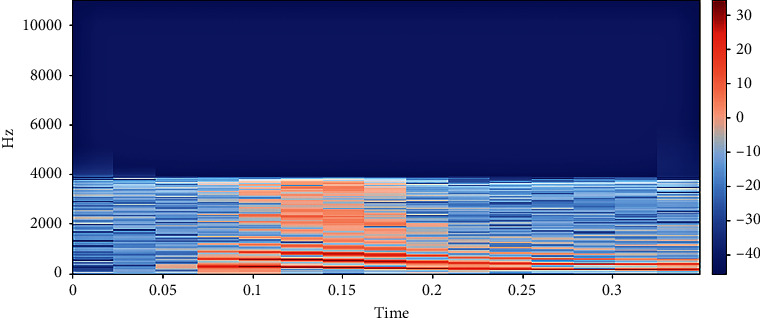
STFT representation of audio data.

*w*(*n*) stands for the analysis window [29], and it is assumed to be non-zero.  Figure 2 represents extracted STFT features.

### 3.2. One-Hot Encoding Method

The proposed method in this work used one-hot encoding as a preprocessing step. The method is applied to the categorical data variables to convert them to a form that is appropriate for ML algorithms to perform an improved task of classification. This involves first mapping the categorical variables to integer values. Then each of the integer values is represented as a binary vector, i.e., all are 0's except for the index integer which is 1.

**Figure 3:**
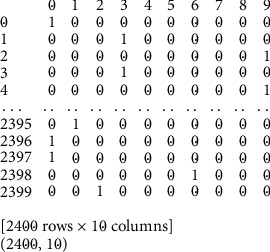
A sample output showing one-hot encoding of the audio data.

The conversion to this form is very necessary because many ML algorithms cannot work with the categorical data directly, it must first be converted to numbers.  Figure 3 shows how each category value is transformed into a new column and assigned a “1” or “0” value, which is a notation for true/false. One-hot encoding of the audio data generates a target class label which was used as input into the proposed model.

### 3.3. Optimization Techniques

#### 3.3.1. Stochastic Gradient Descent

Gradient descent is a method for minimizing a function *J*(*θ*), where *J* is the loss function and *θ* ∈ *R*^*n*^ is the model's parameter vector. To minimize *J*(*θ*), one has to calculate the gradient ▽*J*(*θ*) with respect to the parameter *θ*. Then the parameter *θ* is updated as follows:

(3) $$\begin{array}{c} \theta =\theta -\eta •▽J\left(\theta \right)\text{,} \end{array}$$

where the learning rate *η* controls the size of the steps to reach a local minimum. Formula (3) represents the steepest descent (batch-gradient descent) algorithm for minimizing *J*(*θ*).

For each training example *x*(*i*) and label *y*(*i*), SGD [30] executes an update of the parameter as

(4) $$\begin{array}{c} \theta =\theta -\eta •▽J\left(\theta ;x\left(i\right);y\left(i\right)\right)\text{.} \end{array}$$

SGD was developed to overcome the pitfalls of batch-gradient descent. SGD is faced with the challenge of having to choose an appropriate learning rate, to avoid shifts at the point of convergence.

#### 3.3.2. Adam Optimization Algorithms

Adam [31], is a dynamic method for stochastic optimization that demands only first-order gradients with minimal memory requirement. Adam shows an edge over SGD by combining two other extensions of SGD; adaptive gradient (AdaGrad) [32], and RMSProp [33].

Suppose, we want to solve an optimization problem of the formula as

(5) $$\begin{array}{c} \text{Minimize}f\left(x\right)\text{,} \end{array}$$

where *f*(*x*) is a differentiable stochastic scalar function of the parameter *x*. The underlying idea of the Adam optimization algorithm applied to the above problem is to minimize the expected value, *E*[*f*(*x*)], of the function *f*(*x*). Suppose, *f*_1_(*x*), *f*_2_(*x*),…, *f*_*T*−1_(*x*), *f*_*T*_(*x*) are the functional values of the stochastic function *f*(*x*) at successive time steps 1, 2,…, *T* − 1, *T*. The stochasticity could be due to the evaluation of *f*(*x*) at random subsamples (minibatches) of data points. The gradient of *f*(*x*) at time step *t* is given by *g*_*t*_=∇_*x*_*f*_*t*_(*x*).

The estimate of the first moment (mean) represents the moving average of the gradient. On the other hand, the estimate of the second moment (variance) represents the moving average of the squared gradient. Let *m*_*t*_ be the first moment and *v*_*t*_ the second moment. Then the Adam algorithm computes the first order of momentum (the bias-corrected estimate of *m*_*t*_) as

(6) $$\begin{array}{c} \hat{m_{t}}=\frac{m_{t}}{1-\beta_{1}^{2}}\text{,} \end{array}$$

and the second order of momentum (the bias-corrected estimate of *v*_*t*_) as

(7) $$\begin{array}{c} \hat{v_{t}}=\frac{v_{t}}{1-\beta_{2}^{2}}\text{,} \end{array}$$

*β*_1_, *β*_2_∈ [0, 1] as in equations (6) and (7) are the hyperparameters that control the exponential decay rates of moving averages. For the equations (6), (7), *β*_1_ = 0.9, *β*_2_ = 0.999.

The Adam optimization algorithm used for this study permits the network to achieve high accuracy by regulating the network's weights through an adaptive moment gradient change.

### 3.4. Random Forest Classifier

RF is a supervised ML algorithm that is used widely in classification and regression tasks. It is derived from the concept of ensemble learning, which involves the combination of various classifiers to resolve complicated problems and improve the model performance.

RF and other ensemble methods do not need as much preprocessing as some other methods. RF consists of multiple decision trees, each of which output a prediction. It is often said that in a given forest, more trees make for more robustness. RF creates decision trees through the selection of data samples randomly to get the prediction from each of the trees and then arrive at the best result using balloting [34].

In RF, each decision tree, otherwise known as the base learner, can benefit from a random subset of feature vectors [35]. Consequently, the feature vector is described in the following formula:

(8) $$\begin{array}{c} x=\left(x_{1},x_{2},\ldots ,x_{n}\right)\text{,} \end{array}$$

which is an n-dimensional vector. Let *L*(*Y*, *f*(*x*)) be the loss function. The main objective is to find the function *f*(*x*) that predicts the parameter *Y*.

The goal of the loss function is to minimize the expected value of the loss. Squared error loss and zero-one loss are common choices in regression and classification applications. They are defined in (9) and (10), respectively, [36].

(9) $$\begin{array}{c} L\left(Y,f\left(x\right)\right)=\left(Y-f{\left(x\right)}^{2}\right)\text{,} \end{array}$$

(10) $$\begin{array}{c} L\left(Y,f\left(x\right)\right)=1\left(Y\neq f\left(x\right)\right)\text{,} \end{array}$$

The steps in implementing the RF algorithm are as follows:

- Step 1—First, choose random samples from a given dataset.
- Step 2—Here, the algorithm builds a decision tree for each sample. A prediction outcome is computed from each of the decision trees.
- Step 3—This step performs voting for each of the predicted results.
- Lastly, the final predicted result will be selected as the most voted prediction.

**Figure 4:**
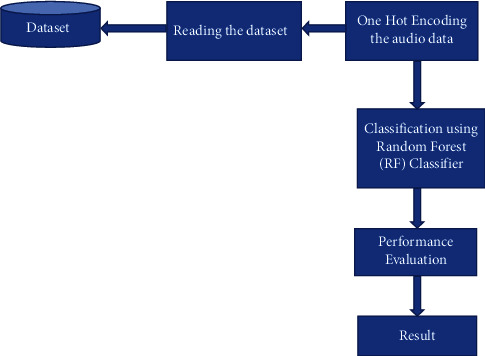
Random forest flowchart.

**Figure 5:**
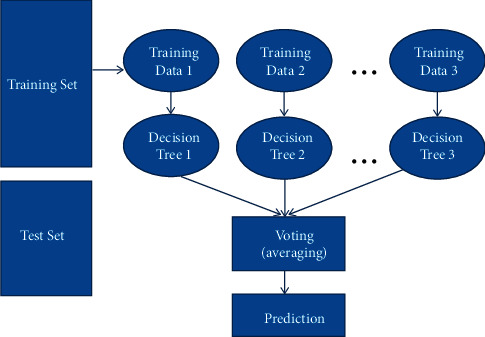
Summary of the workings of the random forest.

RF was implemented on the proposed model using an RF classifier represented as ‘CLF'. Here we set the number of trees in the forest to 100, which is default of n_estimators, while the maximum_depth is set to 5. This implies that the number of decision trees is 100. Then the ‘CLF' is fit to X_train and y_train, respectively, to train the model on the data. The model's accuracy when trained with the RF classifier showed a validation accuracy of 73.67%. The proposed RF algorithm and the corresponding flowchart are described in Algorithm 1 and Figure 4, respectively.  Figure 5 explains the working of the RF algorithm.

### 3.5. Gradient Boosting Classifier

The gradient boosting (GB) [37] classifiers are groups of ML algorithms that merge numerous weak models to produce a stronger predictive model. It is a concept from ensemble learning for solving regression and classification problems. GB combines several decision trees on subparts of the same dataset to form a stronger predictive model.

**Figure 6:**
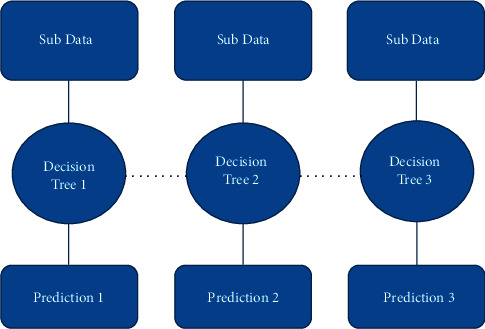
The architecture of gradient boosting.

It integrates multiple machine learning models (mainly decision trees) and every decision tree model gives a prediction. Decision trees are used as the weak learners in GB. Decision trees solve the problem of ML by converting the data into a tree representation. If we align all the decision trees in a successive order, then it can be said that each subsequent model would minimize errors in the prior decision tree model. For a better understanding of the statements above, Figure 6 was used to illustrate.

The first step in GB is to create an initial constant prediction value *F*_0_, where

(11) $$\begin{array}{c} F_{0}\left(x\right)={arg}_{\gamma }\;\min\overset{n}{\underset{i=1}{\sum }}L\left(y_{i},\gamma \right)\text{,} \end{array}$$

where *L* is the loss function, *γ* is the predicted value. Since the target column is continuous, our loss function will be

(12) $$\begin{array}{c} L=\frac{1}{n}\overset{n}{\underset{i=1}{\sum }}L{\left(y_{i},\gamma \right)}^{2}\text{.} \end{array}$$

Here *y*_*i*_ is the observed value, and *γ* is the predicted value. There is a need to find the least value of *γ* that minimizes the loss function.

**Figure 7:**
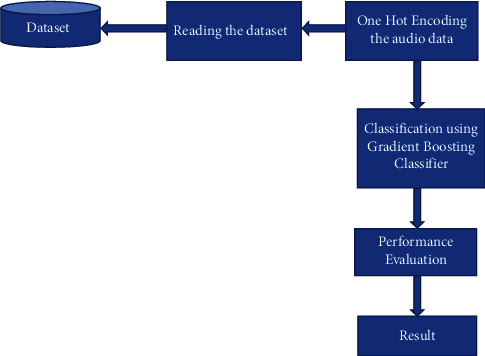
Gradient boost flowchart.

The proposed GB algorithm is defined in Algorithm 2 with the corresponding flowchart as represented in Figure 7. The parameters that were used for the GB classification in this work are defined in Table 1.

### 3.6. The Proposed Deep Feedforward Neural Network Architecture

Deep neural networks (DNNs) have become a fundamental part of state-of-the-art ASR systems [38]. The DNN-based classification as proposed in the study enforced acoustic attributes that are plucked from the raw speech data [39]. In a feedforward neural network, information always travels in one direction [40]. There are no feedback connections and no cycles or loops in the network.

The proposed DFNN model used dense sequential fully connected layers that consist of three hidden layers with 256, 128, and 128 dimensions, respectively. The input and output layers are 1025 and 10 dimensions, respectively. In the first layer, the input layer is of 1025 dimensions whereas the input for the second dense layer is the output of the first layer, which is 256 dimensions. The third layer is related, the model instinctively considers the input dimension to be the same as the output of the last layer, which is 256. The last layer also known as the output layer with 10 dimensions represents 10 classes.

Hyperbolic tangent ( tanh ) activation was used at each level of the network layer except for the output layer. However, the output layer used the softMax activation function. The softMax activation function is often used in deep learning models as the last activation function of the neural network (NN) to regulate the network's output against the predicted output classes. The tanh activation function was also chosen due to its nonlinearity. The output of tanh is between the range of −1 to +1. Like the sigmoid, tanh in addition has a dispersing inclination issue. Tanh is also zero-centred, which enables modeling of inputs with strongly negative, neutral, and positive values.

The DNN's top layer is made up of nodes that employ the softMax activation function [39]. The function permits the DNN to produce class probabilities for each node which sums up to 1.

(13) $$\begin{array}{c} P\left(\left.Y=i\right|x,W,b\right)={\text{softmax}}_{i}\left(W_{x}+b\right)\\ =\frac{e^{W_{i}x+bi}}{\sum_{j}e^{W_{j}x+b_{j}}}\text{,} \end{array}$$

*Y* represents the target mixtures, while *W* and, *b* represents the weight matrix and bias vector, respectively.

(14) $$\begin{array}{c} l\left(\theta =W,b,D\right)=-\overset{\left|D\right|}{\underset{i=0}{\sum }}\log\left(P\left(\left.Y=y_{i}\right|x_{i},W,b\right)\right)\text{.} \end{array}$$

The model proposed here used categorical cross-entropy loss, which specifies multiple classes. Hence, it is a loss function for multiclass classification tasks. They are used for optimizing classification models during training, to reduce loss function. Cross entropy loss is a key factor in deciding how many epochs will be used for a particular model.

**Figure 8:**
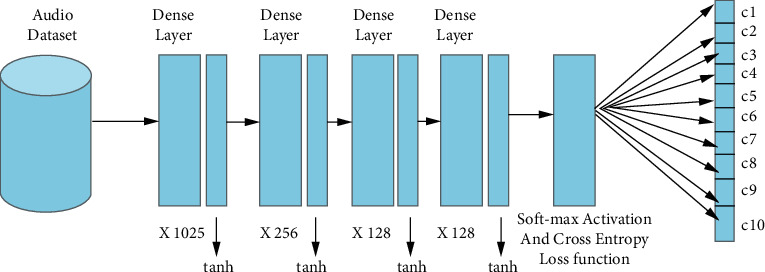
Architectural diagram for the neural network method.

**Figure 9:**
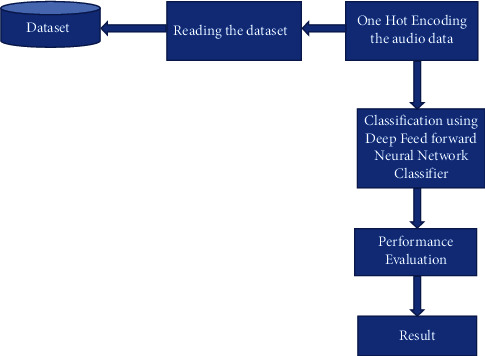
Deep feedforward neural network flowchart.

Adam and SGD are the optimization algorithms for minimizing errors in the proposed DFNN. The results of the two algorithms were compared, and Adam showed better accuracy than SGD. The proposed DFNN structure is represented in Figure 8, whereas the algorithm for the proposed DFNN model is illustrated in Algorithm 3. The flowchart for the proposed DFNN model is illustrated in Figure 9.

## 4. Experimental Results

### 4.1. Dataset

The dataset used for the model's training and validation is a well-founded freely accessible dataset, a collaboration that works with Pannous [41] to improve speech recognition. The dataset is from the Librosa library [42]. It consists of isolated digits with a total of 2400 different audio files in a WAV format for the model training. For the training of each of the proposed models, a training-validation data split of 75%–25% was used. The validation accuracy is fitted into the model's training output. STFT features were used as input and the audio class as the target label in the proposed model.

The RF technique was the first to be implemented in the work using an RF classifier represented as ‘CLF'. The hyperparameter tuning was set up with the number of decision trees = 100, which is the default of n_estimators, while the maximum depth was set to 5. The ‘CLF' was fitted to the X_train and y_train, respectively, to train the model on the data. After training, the result of the RF classifier showed a validation accuracy of 73.67%.

**Figure 10:**
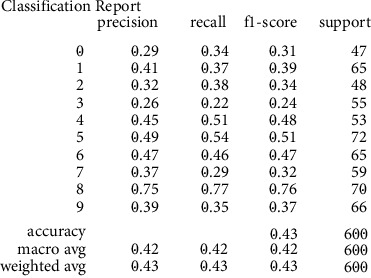
A sample screenshot showing results of each digit's precision, recall, and f1-score.

The model was trained next using the GB classifier with parameters set for n_estimators, max_features, max_depth, learning_rate, and random_state of values 20, 2, 5, 0.05, and 0, respectively. The learning rate was adjusted to 0.075, 0.1, 0.25, 0.5, 0.75, and 1 during training. The best validation accuracy of 81.80% on the training set and 49.00% on the validation set for a 0.75 learning rate were achieved. A sample screenshot showing the results of each digit's precision, recall, and f1-score were as shown in Figure 10.

**Figure 11:**
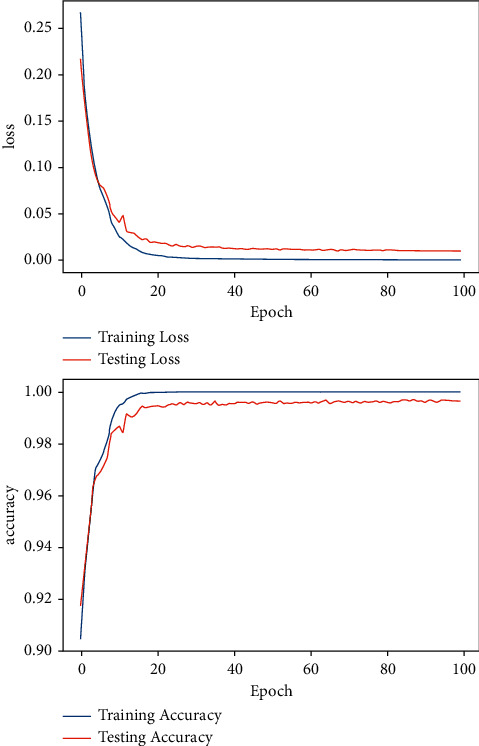
Model accuracy and loss curve diagram using the Adam optimization algorithm.

**Figure 12:**
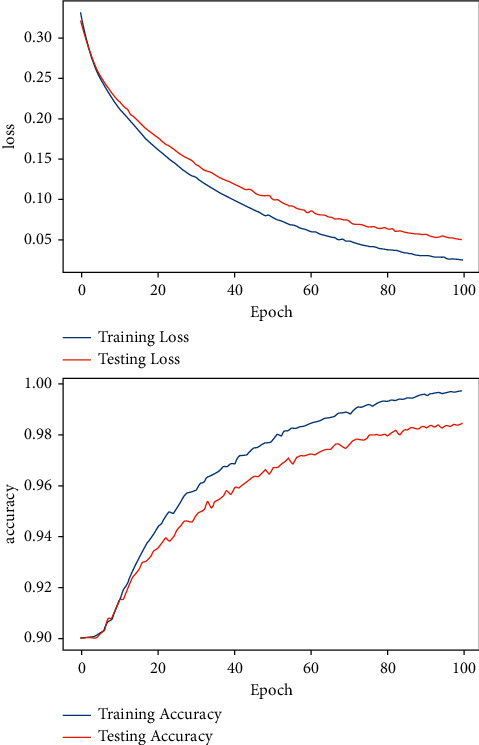
Model accuracy and loss curve diagram using the SGD optimization algorithm.

For DFNN training, the epoch size was set initially to 20 epochs using the Adam optimization algorithm, and increased later to 30, 50, and 100 epochs, respectively. The same number of epoch sizes were replicated for SGD optimization algorithm training. The accuracy of Adam and SGD optimization algorithms for the various epoch sizes; 20, 30, 50, and 100 were calculated and compared. Both Adam and SGD optimization algorithms showed the best accuracy for 100 epochs, as demonstrated in Figures 11 and 12.  Figure 11 shows the accuracy and the loss curve diagram of the model's performance for 100 epochs using Adam optimization algorithms. The model has achieved a validation accuracy of 99.65% and a minimal validation loss of 0.25%.  Figure 12 is the accuracy and loss curve diagram of the model for 100 epochs using the SGD optimization algorithm. The SGD result has shown a validation accuracy of 98.42% and a validation loss value of 0.54%.

Table 2 shows the accuracy comparison of the different ML classification methods used in training the dataset. In Table 2, it was noticed that the DFNN technique exhibited a validation accuracy of 99.65% compared to the other classification methods. The model's performance was compared with some traditional classifiers [22] such as SVM, KNN, and RF on the same dataset. The model proposed in this work attained 99.65% accuracy compared to the conventional approach, as demonstrated in Table 3.

## 5. Discussion

Research on speech classification is still an open issue as a result of the limitations of ASR systems. Recognition of isolated words/digits is practically arduous. A classification technique that involved three techniques; DFNN with hyperparameter optimization techniques, an ensemble method, i.e., RF, and a regression method, i.e., GB, was proposed in this study for the classification of spoken English digit data with the primary objective of determining the best method among them.

Figure 5 shows the working of the RF algorithm. For a better understanding of the RF algorithm, knowledge of the decision tree algorithm is vital. The validation accuracy for the RF classifier after training shows a result of 73.67% accuracy, which is not high. This suggests that more decision trees should be created since the greater number of trees in the forest results in greater accuracy while overfitting is avoided.

Figure 6 shows the architecture of the GB. The GB's performance was compared for different learning rates; lr_list = (0.05, 0.075, 0.1, 0.25, 0.5, 0.75, 1), where ‘lr' represents the learning rate. Training the proposed model with variable learning rates achieved 81.80% on the training set and 49.00% on the validation set for a 0.75 learning rate. This is an indication that the GB was overfitting the training dataset which affects the accuracy.

Figure 8 shows the architectural diagram of the proposed DFNN model. The model was trained first using the Adam optimization algorithm and retrained using the SGD optimization algorithm using variable epoch sizes. Increasing epoch size helps in enhancing the model's network accuracy. Epoch performs an essential function in the network training of a model [43]. The total amount of epochs to be applied in network training would help to determine whether the data is overtraining or not.

**Figure 13:**
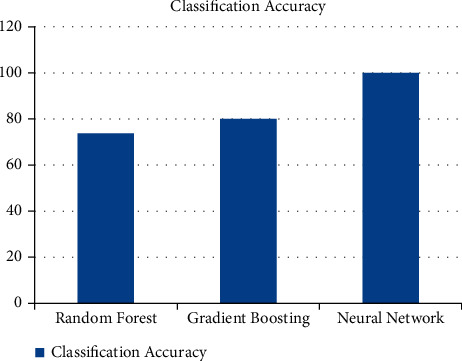
A bar chart for comparing the classification accuracy of the machine learning algorithms.

The performance evaluation in Table 3 suggests that the proposed deep feedforward method is optimal for spoken digit classification. A summary of the performance of the ML algorithms used for this work is depicted as a bar chart in Figure 13.

## 6. Conclusion

Classification of spoken English digit data was conducted using DL methods; ensemble, regression, and a DFNN method with hyperparameter optimization algorithms. STFT feature extraction and a one-hot encoding was implemented on spoken digit data to produce the STFT features as input and the audio class as the target label in the proposed model. Classification results of the training have shown that the DFNN model outperformed the RF and GB models with the validation accuracy of 99.65% compared to the 73.67% and 79.70% accuracy of RF and GB, respectively. Hence, the DFNN model is an efficient approach for the classification of spoken English digit data.

## Figures and Tables

**Algorithm 1:**
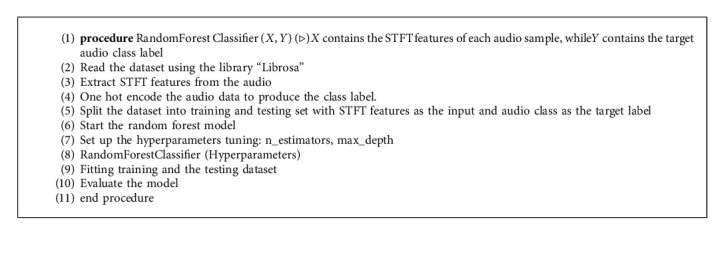
The Random Forest Classification Model.

**Algorithm 2:**
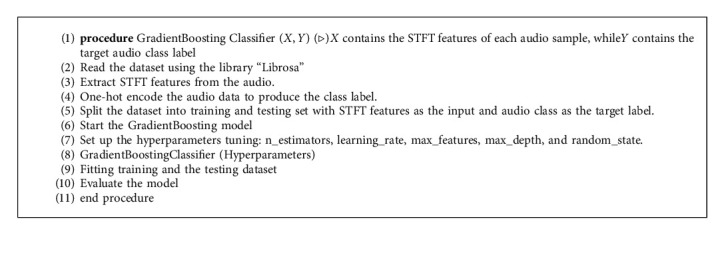
The GradientBoosting Classification Model.

**Algorithm 3:**
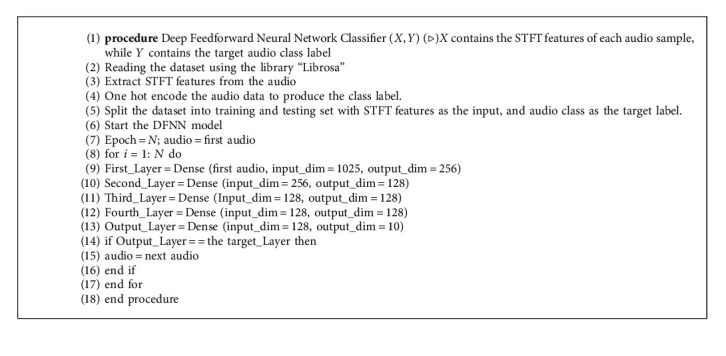
The DFNN Classification Model.

**Table 1:** Parameters used for the GB Classification.

Parameters	Denoted As	Value
Number of estimators	n_estimators	20
Maximum features	max_features	2
Maximum depth	max_depth	5
Learning rate	learning_rate	0.05
Random state	random_state	0

**Table 2:** Accuracy comparison of the various classification algorithms.

Algorithm	Random forest	Gradient boosting	Deep feedforward neural network
Accuracy	73.67%	79.70%	99.65%

**Table 3:** Accuracy Comparison of the Various classification Algorithms.

Method	Dataset	Features	Classifier	Accuracy
Supervised learning	Spoken English digit	MFCC	SVM + KNN + RF	97.50%
The proposed model	Spoken English digit	STFT	RF + GB + NN	99.65%

## Data Availability

The data used are available from the corresponding author upon request.

## References

[1] Nasreen P. N., Kumar A. C., Nabeel P. A. Speech analysis for automatic speech recognition.

[2] Jurafsky D., Martin J. H. (2020). *Speech and Language Processing*.

[3] Nguyen Q. T., Bui T. D. (2016). Speech classification using SIFT features on spectrogram images. *Vietnam Journal of Computer Science*.

[4] Goodfellow I., Bengio Y., Courville A., Bengio Y. (2016). *Deep learning*.

[5] Silva D. F., de Souza V. M. A., Batista G. E. A. P. A. (2013). A comparative study between MFCC and LSF coefficients in automatic recognition of isolated digits pronounced in Portuguese and English. *Acta Scientiarum. Technology*.

[6] Kopparapu S. K., Rao P. V. S. (2004). Enhancing spoken connected-digit recognition accuracy by error correction codes—a novel scheme. *Sadhana*.

[7] Nimje K., Shandilya M. (2011). Automatic isolated digit recognition system: an approach using HMM. *Journal of Scientific and Industrial Research*.

[8] Silva D. F., de Souza V. M. A., Batista G. E. A. P. A., Giusti R. (2012). Spoken digit recognition in Portuguese using line spectral frequencies. *Ibero-American Conference on Artificial Intelligence*.

[9] Alotaibi Y. A. (2005). Investigating spoken Arabic digits in speech recognition setting. *Information Sciences*.

[10] Shyu R.-C., Wang J.-F., Lee J.-Y. (2000). Improvement in connected Mandarin digit recognition by explicitly modeling coarticulatory information. *Journal of Information Science and Engineering*.

[11] Tyagi K., Tyagi K. (2015). A comparative analysis of optimization techniques. *International Journal of Computer Application*.

[12] Oruh J., Viriri S. (2021). Deep learning with optimization techniques for the classification of spoken English digit. *International Conference on Computational Collective Intelligence*.

[13] Sharmin R., Rahut S. K., Huq M. R. (2020). Bengali spoken digit classification: a deep learning approach using convolutional neural network. *Procedia Computer Science*.

[14] Megala D. S. S., Padmapriya R., Jayanthi B., Suganya M. (2019). Detection and classification of speech pathology using deep learning. *International Journal of Scientific & Technology Research*.

[15] Martins R., Gomes M., Almeida J. J., Novais P., Henriques P. Hate speech classification in social media using emotional analysis.

[16] Lech M., Stolar M., Best C., Bolia R. (2020). Real-time speech emotion recognition using a pre-trained image classification network: effects of bandwidth reduction and companding. *Frontiers of Computer Science*.

[17] Gu Y., Li X., Chen S., Zhang J., Marsic I. (2017). Speech intention classification with multimodal deep learning. *Adv Artif Intell*.

[18] Mamyrbayev O., Mekebayev N., Turdalyuly M., Oshanova N., Medeni T. I., Yessentay A. (2019). Voice identification using classification algorithms. *Intelligent System And Computing*.

[19] Zada B., Ullah R. (2020). Pashto isolated digits recognition using deep convolutional neural network. *Heliyon*.

[20] Dawodi M., Baktash J. A., Wada T., Alam N., Joya M. Z. Dari speech classification using deep convolutional neural network.

[21] Marcolla F. M., de Santiago R., Dazzi R. L. S. Novel lie speech classification by using voice stress.

[22] Maddimsetti Srinivas K. M., Ashok G. L. P. (2019). Spoken English digit classification using supervised learning. *International Journal of Research in Signal Processing, Computing & Communication System Design*.

[23] Ahamed S., Weiler G., Boden K. (2021). Deep neural network driven speech classification for relevance detection in automatic medical documentation. *Studies in Health Technology and Informatics*.

[24] Sánchez-Hevia H. A., Gil-Pita R., Utrilla-Manso M., Rosa-Zurera M. (2022). Age group classification and gender recognition from speech with temporal convolutional neural networks. *Multimedia Tools and Applications*.

[25] Jena P. M., Nayak S. R. (2018). Angular symmetric Axis constellation model for off-line Odia handwritten characters recognition. *International Journal of Advances in Applied Sciences*.

[26] Sharma N., Gupta S., Mehta P. (2022). Offline signature verification using deep neural network with application to computer vision. *Journal of Electronic Imaging*.

[27] Sethy A., Patra P. K., Nayak S. R. (2022). A hybrid system for handwritten character recognition with high robustness. *Traitement du Signal*.

[28] Oruh J., Viriri S., Renault É., Boumerdassi S., Mühlethaler P. (2021). Spectral analysis for automatic speech recognition and enhancement. *Machine Learning for Networking. MLN 2020. Lecture Notes in Computer Science*.

[29] Krishnan S., Krishnan S. (2021). advanced analysis of biomedical signals. *Biomedical Signal Analysis for Connected Healthcare*.

[30] Bottou L. (2010). Large-scale machine learning with stochastic gradient descent. *Proceedings of COMPSTAT’2010*.

[31] Kingma D. P., Ba J. (2014). Adam: a method for stochastic optimization. https://arxiv.org/abs/1412.6980.

[32] Duchi J., Hazan E., Singer Y. (2011). Adaptive subgradient methods for online learning and stochastic optimization. *Journal of Machine Learning Research*.

[33] Tieleman T., Hinton G. (2012). Lecture 6.5-rmsprop, Coursera: Neural Networks for Machine Learning.

[34] Breiman L. (2001). Random forests. *Machine Learning*.

[35] Lee T.-H., Ullah A., Wang R. (2020). Bootstrap aggregating and random forest. *Macroeconomic Forecasting in the Era of Big Data*.

[36] Savargiv M., Masoumi B., Keyvanpour M. R. (2021). A new random forest algorithm based on learning automata. *Computational Intelligence and Neuroscience*.

[37] Friedman J. H. (2002). Stochastic gradient boosting. *Computational Statistics & Data Analysis*.

[38] Hinton G., Deng L., Yu D. (2012). Deep neural networks for acoustic modeling in speech recognition: the shared views of four research groups. *IEEE Signal Processing Magazine*.

[39] Saleem M. M. (2014). *Deep Learning for Speech Classification and Speaker Recognition*.

[40] Zell A. (1994). *Simulation neuronaler netze*.

[41] Pannous.Github (2014). Pannous/TensorFlow-speech-recognition. http://github.com/pannous/tensorflow-speech-recognition.

[42] McFee B., McVicar M., Raffel C. (2015). Librosa: v0.4.0.Zenodo. https://zenodo.org/record/18369#.YxurLj1BzIU.

[43] Afaq S., Rao S. (2020). Significance of epochs on training a neural network. *International Journal of Scientific and Technology Research*.

